# Long Time from Diagnosis to Surgery May Increase Postoperative Complication Rates in Elective CD Intestinal Resections: An Observational Study

**DOI:** 10.1155/2018/4703281

**Published:** 2018-04-23

**Authors:** Paulo Gustavo Kotze, Daniela Oliveira Magro, Carlos Augusto Real Martinez, Antonino Spinelli, Takayuki Yamamoto, Janindra Warusavitarne, Claudio Saddy Rodrigues Coy

**Affiliations:** ^1^Colorectal Surgery Unit, Cajuru University Hospital, Pontifical Catholic University of Paraná (PUCPR), Curitiba, PR, Brazil; ^2^Colorectal Surgery Unit, University of Campinas (UNICAMP), Campinas, SP, Brazil; ^3^Department of Colorectal Surgery, Humanitas Research Hospital, Rozzano, Italy; ^4^IBD Centre, Yokkaichi Hazu Medical Centre, Yokkaichi, Japan; ^5^Department of Colorectal Surgery, St. Mark's Hospital, Harrow, UK

## Abstract

**Background:**

There is lack of data analyzing short-term postoperative complications and time from diagnosis to surgery in Crohn's disease (CD).

**Aim:**

To compare complication rates after elective abdominal operations in CD patients with different durations of disease.

**Methods:**

Retrospective observational study with CD patients who submitted to elective intestinal resections. Patients were allocated in 2 groups according to time to surgery (TS) in less or more than 5 years. Short-term postoperative complications were analyzed and compared between the 2 groups, and binary logistic regression analysis was performed to check for significant variables.

**Results:**

123 patients were finally included, 77 with TS > 5 years (62.6%) and 46 with TS < 5 years (37.4%). Patients with TS > 5 years had higher rates of overall surgical complications (*p* = 0.011), reoperations (*p* = 0.003), surgical site infections (*p* = 0.014), anastomotic dehiscence (*p* = 0.021), abdominal abscesses (*p* = 0.021), and overall medical complications (*p* = 0.019). On logistic regression, the single significant variable was the confection of stomas (OR: 3.203; 95% CI: 1.011–10.151; *p* = 0.048).

**Conclusions:**

Patients with longer time to surgery showed a significant increase in overall medical and surgical postoperative early complications after elective intestinal resections.

## 1. Introduction

Crohn's disease (CD) has a progressive natural course that evolves from luminal inflammation to fibrosis and stenosis of bowel segments, potentially culminating in perforation, abscesses, and fistulas [1]. Surgery is an important tool in the management of the disease and is often used in the stenotic and penetrating phenotypes of the disease [2]. Clearly, longer time from diagnosis to surgery tends to lead to a more complex disease phenotype, sometimes limiting the action of minimally invasive surgical procedures due to extensive masses and internal or external fistulas and involvement of adjacent healthy small bowel.

There is controversy if biological agents are changing the natural history of surgery in CD. There seems to be a reduction in surgical procedures when biological therapy is appropriately timed, in population-based studies [3]. Other studies suggest that there can be a delay in the indication for surgery after persistent medical therapy, leading to significant consequences, such as more extensive procedures owing to increased severity at the time of surgery [4, 5]. Nonetheless, the extension of surgical specimens has not reduced in number based on a study from the Netherlands [6].

Different studies analyzed the long-term course of the disease after early surgery in CD. A study from Italy evaluated the disease course in CD patients comparing early (at the time of diagnosis) with delayed surgery [7]. They found that cumulative clinical recurrence was lower in the early surgery group (hazard ratio (HR) = 0.57; 95% CI: 0.35 to 0.92; *p* = 0.02). However, there was no difference between the groups in surgical recurrence and the need for immunosuppressants. A similar study from Hungary demonstrated that early limited surgery leads to a reduction in the need for steroids and biologics over time, but again, no impact in surgical recurrence was seen [8].

A retrospective study from Portugal compared the outcomes of patients that had surgery less than 6 months from diagnosis with those of patients that had only immunosuppressants (no indication for surgery). The rates of unfavourable outcomes were similar between the groups, and patients with early surgery had more reoperations over the long-term follow-up [9]. More recently, the LIR!C trial compared early ileocaecal resection with infliximab (IFX) therapy for localized luminal CD [10]. The results demonstrated that surgery was at least as good as IFX in terms of quality of life, demonstrating that laparoscopic resection is also an option for these patients, with low complication rates.

Indeed, there is lack of data regarding the relation between short-term postoperative complications and time to surgery (TS) from diagnosis. Patients with longer TS probably have more severe phenotypes of the disease, with a possible increase in postoperative complications. On the contrary, early indication for surgery after medical failure could lead to less invasive approaches, more limited resections, and possibly lower complication rates.

The aim of this study was at analyzing and comparing complication rates after elective abdominal operations in CD patients with different durations of disease from diagnosis to surgery.

## 2. Methods

### 2.1. Study Design

This was a retrospective and observational study, with CD patients submitted to intestinal resections due to disease complications (stenosis or fistulas) or failure to medical treatment, from 2 different IBD referral units, in a period of 6 years.

### 2.2. Inclusion and Exclusion Criteria

Patients over 18 years of age, with an established diagnosis of CD with clinical, imaging, endoscopic, and histological criteria, submitted to any elective abdominal surgical procedure that included intestinal resection in any segment, were included in the analysis. We excluded from the analysis patients submitted to other procedures without intestinal resection (such as strictureplasties or diverting stomas) and those operated in the emergency setting. These patients were excluded to reduce any potential bias arising from the premise that complication rates might be lower in strictureplasties [11] and higher in emergency operations.

### 2.3. Variables Analyzed

Baseline demographic characteristics were analyzed: age at diagnosis and at surgery, gender, time to surgery (TS), and smoking status.

The phenotype of each patient was described according to the Montreal classification (perianal disease included). The main operative characteristics were also evaluated: indication for surgery (medical failure, stenosis, or fistulizing disease), type of procedure according to the location, and approach (conventional or laparoscopic). The presence of hypoalbuminemia (albumin < 3.0 mg/dL) and preoperative medication (steroids, immunomodulators, or/and biological agents) were also part of the study protocol.

Early postoperative surgical and medical complications (up to 30 days after the surgical procedure) were subsequently evaluated. We considered surgical complication as any deviation from the normal postoperative period that needed intervention (abdominal abscess, anastomotic dehiscence, surgical site infection (SSI), or reoperation). Medical complications were defined as any postoperative occurrence other than those related to the surgical site. Medical complications relevant to this study were pneumonia, urinary tract infections, overall infections, and any other complication that did not require surgery for treatment (e.g., ileus or pancreatitis). All variables were defined before the data collection.

Preoperative exposure to biologics was defined as treatment with anti-TNF agents (infliximab (IFX) or adalimumab (ADA)) up to 8 weeks prior to the surgical procedure. Prior treatment with steroids and thiopurines was also recorded. Previous exposure to steroids was defined as a dose of 20 mg of prednisone up to 6 weeks before the surgical procedures, according to the ECCO (European Crohn's and Colitis Organisation) guidelines [2].

The protocols with the variables previously described were retrospectively completed, after electronic chart review. The postoperative medical and surgical complications were then identified and compared between the 2 different groups.

### 2.4. Group Definition

After patient identification and selection, patients were allocated to 2 groups, based on time to surgery (TS). We defined TS as the period from confirmation of diagnosis to the first elective surgical procedure. Patients were allocated to two groups on the basis of an arbitrary cutoff of 5 years (TS > 5 years and TS < 5 years). As TS was measured in months, patients with precisely 60 months of TS were included in the TS < 5 years group.

### 2.5. Statistical Analysis

Data analyses were performed using SPSS v.20.0 (SPSS Inc., Chicago, IL). The results were showed as mean ± SD for continuous variables and proportions for categorical variables. Dichotomous variables were compared, between groups, using Pearson's chi-square test or Fischer's exact test. To compare the results obtained in two groups, the Student's *t*-test method was used.

Multivariate binary logistic regression analysis was performed to determine risk factors for clinical and surgical complications and groups (TS > 5 years and TS < 5 years). The odds ratios (ORs) were presented with 95% confidence interval (95% CI). All covariates included within logistic regression were categories (abdominal abscess, anastomotic dehiscence, surgical site infection, reoperation, bowel obstruction, stomas, pneumonia, urinary tract infections, hypoalbuminemia, previous steroids, and death). The method applied was “enter.” Differences with *p* < 0.05 were considered statistically significant.

### 2.6. Ethical Considerations

The study was approved by the board of the ethical committees of both institutions, according to protocol 63324/2012 and CAAE 03687612.8.1001.5404, from the ministry of health website “*Plataforma Brasil.*”

## 3. Results

Initially, 144 patients were selected from the database and had their files accessed. From those, 21 were excluded (7 patients with strictureplasties, 2 patients with diverting stomas without associated resection, and 12 due to emergency operations). A total of 123 patients were finally included in the study.

The baseline characteristics of the patients are described in detail in Table 1. The majority of patients (62.6%) had TS > 5 years (mean 153.44 months ± 60.2). The group with TS < 5 years had a mean of 31.9 ± 17.0 months. Patients in the TS > 5 years group also had more Montreal A2, L2, L3, and B3 disease phenotypes, as well as perianal fistulizing CD. Patients with TS > 5 years also had a higher prevalence of hypoalbuminemia before surgery and more stoma confection as a surgical approach and more patients needed surgery for stenotic disease in this group. More open procedures were performed in the TS < 5 years group.

**Figure 1:**
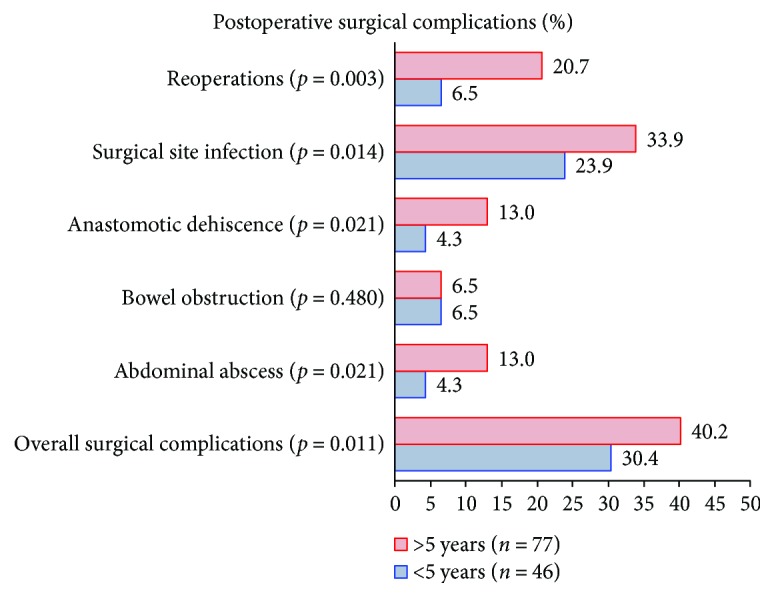
Postoperative surgical complications between the groups. Higher prevalence of reoperations, surgical site infection, anastomotic dehiscence, abdominal abscess, and overall complications were observed in patients with TS > 5 years. No difference was observed in bowel obstruction.

Figure 1 demonstrates postoperative surgical complications between the 2 different groups. Patients with delayed surgery, with TS > 5 years, had significantly higher rates of overall surgical complications (40.2% versus 30.4%; *p* = 0.011), reoperations (20.7% versus 6.5%; *p* = 0.003), SSI (33.9% versus 23.9%; *p* = 0.014), anastomotic dehiscence (13% versus 4.3%; *p* = 0.021), and abdominal abscesses (13% versus 4.3%; *p* = 0.021) as compared to those with TS < 5 years.

**Figure 2:**
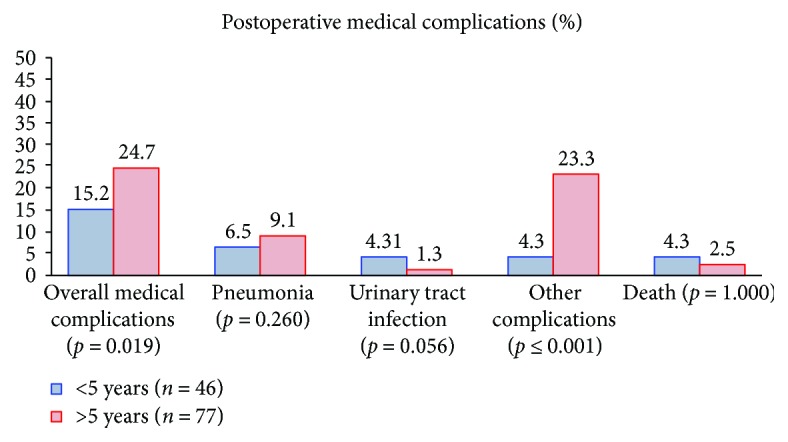
Postoperative medical complications between the groups. Overall complications and other complications were more prevalent in the TS > 5 years group. No statistical difference was seen in pneumonia, urinary tract infection, or mortality between the groups.

Medical postoperative complications were also analyzed and are demonstrated in detail in Figure 2. Overall postoperative medical complications (24.7% versus 15.2%; *p* = 0.019) and other complications (23.3% versus 4.3%; *p* < 0.001), such as infections not related to the surgical site, pancreatitis, and postoperative ileus, were more prevalent among patients with TS > 5 years. There was no statistical difference between the groups in terms of pneumonia, urinary tract infection, or mortality.

On logistic regression analysis, the only identified significant variable was the confection of stomas (OR: 3.203; 95% CI: 1.011–10.151; *p* = 0.048). This data suggests that patients submitted to surgery in the TS > 5 years group had 3 times higher risk of having a stoma, as compared to patients from the TS < 5 years group. All variables from the logistic regression analysis are demonstrated in detail in Table 2.

## 4. Discussion

Progression to bowel damage is an intrinsic characteristic of CD, and this natural evolution can occur despite optimal medical therapy. It is known that surgery plays an important role in the therapeutic armamentarium in CD, but there is still controversy if effective medical treatment, such as biological therapy, can avoid or simply delay the need for surgical intervention due to irreversible bowel damage [3, 5, 12].

There is some evidence that early surgery can lead to a reduction in clinical recurrence, but not surgical recurrence in long term [7, 8]. Currently, there is lack of data on short-term postoperative outcomes in relation to timing of surgery. Longer TS in CD is often associated with worse outcomes and lower response rates to medical therapy [13]. If the same pattern could be observed in surgical therapy in early versus late disease, this needs further investigation, as there are only few studies in the field [4].

Our observational study demonstrated that patients with longer TS (>5 years) had significantly higher rates of overall medical and surgical postoperative complications as compared to patients operated earlier in the disease course. An initial definition from a panel of experts defined early CD as 2 years from diagnosis to initiation of therapy [14]. More recently, the timeframe was reduced to 18 months, as a standard to be used in medical therapy trials [15]. There is currently no definition of early versus late disease for surgical treatment in CD.

The definition of a cutoff of 5 years to divide the groups of our study was arbitrary. The main reason for the specific point of 5 years was to have enough patients in each group, as an attempt to perform the cutoff point at 2 or 3 years would result in a reduced number of patients in the early disease group, with the resultant inability to form meaningful conclusions. Even with our arbitrary definition, the early disease group had a mean of 31.93 months of TS (less than 3 years) as compared to 153.44 months (more than 12 years) in the late disease group, allowing a reasonable validation of the study's conclusion. The definitions of early surgery in other studies have varied from the time of disease diagnosis to 6 months from TS. This highlights the fact that this could not be applied to our study due to the reduced number of procedures in the first year after diagnosis [7, 8].

The exact reason for having poorer outcomes in the longer TS group could only be speculated in an observational study such as this. As expected, patients with TS > 5 years had more penetrating disease (*p* = 0.047), perianal CD (*p* = 0.001), and higher prevalence of preoperative hypoalbuminemia (*p* = 0.003). Moreover, preoperative use of steroids was also more prevalent in this group of patients (39% versus 26.08%), despite not being statistically significant. This could suggest that patients with delayed surgical indication had markers of more severe disease, such as bowel damage with abdominal and perianal penetrating disease also represented by lower albumin levels. These facts per se could justify an increase in postoperative complications in this specific group of patients. As per study design, the groups were not fully homogeneous in the baseline characteristics, as disease severity increases over time.

The increased rates of surgical postoperative complications can also be justified by more difficult procedures in complicated disease in the longer TS group. Clearly, patients operated with penetrating disease, internal and external fistulas associated or not to abscesses, represent more challenging cases and are more prompt to be associated with complications [4]. These phenotypes of the disease represent a consequence of longer TS, with a progressive disease course despite adequate medical therapy. Approximately 50% of the included patients had preoperative use of anti-TNF therapy. This could mean that some patients with fibrotic disease may have had suboptimal indication for biologic treatment and surgery was still required over time.

Patients with stomas, in our study, were 3 times more likely to be operated after 5 years of TS. More reoperations are clearly linked to the increased rates of surgical postoperative complications, mainly anastomotic leaks and abscesses, which were observed. Increased rates of stomas could again represent not only a consequence of more complications but also a different surgical strategy in more severe cases, when diversion in the elective setting represents a safer procedure in patients at a high risk for complications.

Despite the fact that more severe cases were observed in the longer TS group, there was no difference between the groups in terms of laparoscopic approach. On the contrary, more open procedures were performed in the TS < 5 years group. Possibly, higher rates of open procedures would be expected in the late disease group and more laparoscopic operations in the patients with TS < 5 years, but this was not confirmed in our study. This may represent the inherent difficulties faced by the public hospitals in Brazil with limited access to laparoscopic equipment, energy devices, and endoscopic staplers.

Our study has some limitations. Firstly, all biases regarding retrospective chart analysis, from two different referral centers, and the reduced number of patients must be considered. Secondly, the cutoff of 5 years may not be ideal, but even with this cutoff, the association of late disease with higher short-term complications was clear, and possibly, a reduction to 2 or 3 years could increase the differences even further. Another limitation was the exclusion of emergency procedures and strictureplasties. However, there was a limited number of those operations and this possibly did not impact the results. Additionally, the dose of steroids could also not be extracted in the patients that were exposed to these agents. Despite these limitations, our analysis corroborates the few already available evidence that there is an impact of time from diagnosis to elective abdominal surgery on short-term outcomes in CD patients.

In summary, in this observational cohort, patients with longer time from diagnosis to surgery showed a significant increase in overall medical and surgical postoperative early complications after elective intestinal resections. Patients operated late in the disease course have increased risk of reoperations and stomas. Our findings could represent that a delay in surgical indication, despite optimal medical therapy, can impact surgical outcomes. This emphasizes the need for a multidisciplinary team in the management of CD, with intensive surgical participation early in the disease course, not only in obvious complicated cases. More prospective data with early surgery in CD are warranted.

## Figures and Tables

**Table 1:** Baseline characteristics and type of surgery performed in the two groups stratified by TS (< or >5 years of diagnosis).

Variables *n* (%)	TS < 5 years, *n* = 46 (37.4%)	TS > 5 years, *n* = 77 (62.6%)	*p* value
Male, *n* (%)	26 (56.5)	41 (53.2)	0.720
Female, *n* (%)	20 (43.4)	36 (46.7)	
Age at diagnosis, *n* (%)			
A1	13 (28.2)	11 (14.2)	**<0.001** ^∗∗^
A2	24 (52.1)	55 (71.4)	
A3	9 (19.5)	11 (14.2)	
Location of the disease, *n* (%)			
L1	16 (34.7)	17 (22.0)	**<0.001** ^∗^
L2	7 (15.2)	19 (24.6)	
L3	22 (47.8)	41 (53.2)	
L4	1 (2.1)	—	**—**
Disease phenotype, *n* (%)			
B1	5 (10.8)	7 (9.1)	**<0.001** ^∗∗^
B2	26 (56.5)	42 (54.5)	
B3	15 (32.6)	28 (36.3)	
Perianal disease, *n* (%)			
*p*	12 (26.0)	34 (44.1)	**0.001** ^∗∗^
Active smoking, *n* (%)	5 (10.8)	6 (7.7)	0.760
Hypoalbuminemia	09 (19.6)	27 (35.1)	**0.003** ^∗^
Previous steroids, *n* (%)	12 (26.0)	30 (39.0)	0.140
Combo therapy (anti-TNF + immunomodulator), *n* (%)	22 (47.8)	39 (50.6)	0.760
Anti-TNF therapy, *n* (%)	25 (54.3)	46 (59.7)	0.550
Indications for surgery			
Medical therapy failure	3 (6.5)	7 (9.1)	0.206
Fistula	15 (32.6)	22 (28.6)	0.250
Stenosis	28 (60.9)	48 (62.3)	**0.022** ^∗^
Laparoscopic approach	13 (28.2)	24 (31.1)	0.071
Conventional open approach	33 (71.7)	53 (68.8)	**0.031** ^∗^
Enterectomy	16 (34.7)	24 (31.1)	0.206
Ileocaecal resection	24 (52.1)	35 (45.4)	0.152
Segmental colectomy	7 (15.2)	6 (7.8)	0.780
Total colectomy	0	7 (9.1)	**—**
Total proctocolectomy	1 (2.1)	6 (7.7)	0.059
Stomas	04 (8.7)	18 (23.4)	**0.003** ^∗^

^∗^ *p* < 0.05; ^∗∗^*p* < 0.001; TNF: tumor necrosis factor.

**Table 2:** Model of binary logistic regression analysis. Only the confection of stomas was identified as significant. Patients with TS > 5 years had higher risk of stomas with an odds ratio (OR) of 3.203.

Covariant	Odds ratio (95% CI)	*p* value
Reoperations	0.314 (.050–1.981)	0.218
Surgical site infection	1.269 (0.459–3.514)	0.646
Anastomotic dehiscence	0.280 (.038–2.050)	0.210
Bowel obstruction	1.788 (0.260–12.324)	0.555
Abdominal abscess	2.680 (0.228–31.452)	0.433
Hypoalbuminemia	0.401 (0.139–1.155)	0.090
Stomas	3.203 (1.011–10.151)	**0.048**
Previous steroids	0.494 (.194–1.260)	0.140
Pneumonia	4.357 (0.582–32.618)	0.152
Urinary tract infection	5.912 (.379–92.299)	0.205
Death	4.919 (0.275–87.957)	0.279
